# Diffusiophoretic Movements of Polystyrene Particles in a H-Shaped Channel for Inorganic Salts, Carboxylic Acids, and Organic Salts

**DOI:** 10.1021/acs.langmuir.2c01577

**Published:** 2022-09-28

**Authors:** Nicole A. B. Timmerhuis, Rob G. H. Lammertink

**Affiliations:** Soft Matter, Fluidics and Interfaces, MESA+ Institute for Nanotechnology, University of Twente, Post Office Box 217, 7522 NB Enschede, Netherlands

## Abstract

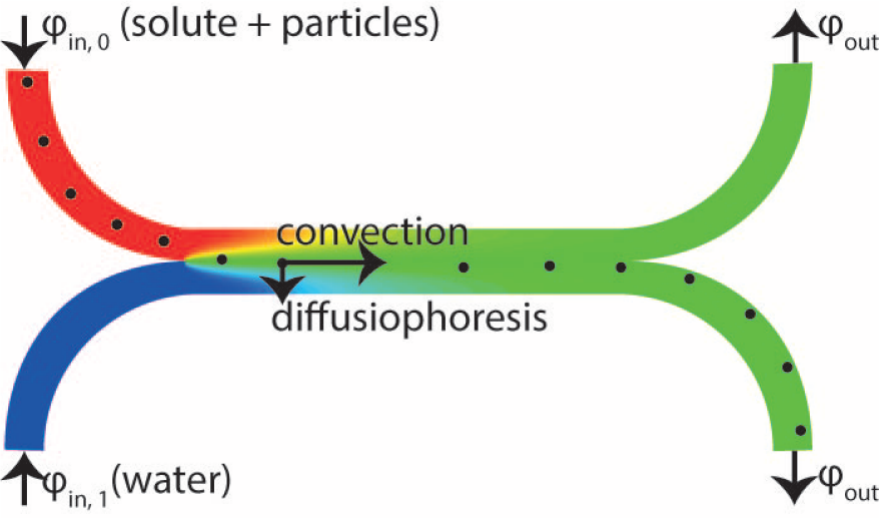

Diffusiophoresis is the movement of particles as a result of a concentration gradient, where the particles can move toward higher concentrations. The magnitude of the movement is largest for the electrolyte solute and depends upon the relative concentration gradient, surface potential, and diffusivity contrast between the cation and anion. Here, diffusiophoresis of ordinary polystyrene particles is studied in a H-shaped channel for different solutes. The experimental results are compared to a numerical model, which is solely based on the concentration gradient, surface potential, and diffusivity contrast. The surface potential of the particles was measured to use as input for the numerical model. The diffusiophoretic movement of the experiments aligns well with the theoretical predicted movement for the inorganic (lithium chloride and sodium bicarbonate) and organic (lithium formate, sodium formate, and potassium formate) salts measured. However, for the carboxylic acids (formic, acetic, and oxalic acids) measured, the theoretical model and experiment do not align because they are weak acids and only partially dissociate, creating a driving force for diffusiophoresis. Overall, the H-shaped channel can be used in the future as a platform to measure diffusiophoretic movement for more complex systems, for example, with mixtures and asymmetric valence electrolytes.

## Introduction

Particles move along a solute concentration gradient as a result of diffusiophoresis,^1^ where the solute interacts with the surface. The solute can either be a non-electrolyte^2^ or an electrolyte,^3^ where the largest phoresis magnitudes were found for electrolyte systems.^4^ Velegol et al.^4^ provided an overview of diffusiophoretic transport in both artificial and natural systems, where diffusiophoresis has a significant impact. Diffusiophoresis can be used to separate colloids from a solution, as shown for different configurations by Shin,^1^ and to improve mixing of colloidal suspensions.^5^ Applications of colloidal separation include water treatment, drug production, disease detection and prevention, personal care products, and food processing.^1^

Diffusiophoresis is a non-equilibrium phenomenon originating from solute–surface interactions in a concentration gradient.^1,3^ We consider a particle in a monovalent electrolyte solution with a charged wall at ζ potential. The particle is submerged in a concentration gradient over much greater distance than the interaction range, where the interaction range is approximated by the Debye layer with thickness κ^–1^. Counterions are attracted toward the surface within the Debye layer, creating a diffusion potential gradient in the bulk. The buildup of diffusion potential results in an electric field, which leads to an electrophoretic flow within the Debye layer of the particle. Simultaneously, an osmotic pressure difference arises within the Debye layer as a result of the concentration difference. This osmotic pressure is balanced by a viscous force, creating a chemiphoretic flow, which propels the particle toward the higher concentration region.

The magnitude of diffusiophoresis depends upon the electrophoretic and chemiphoretic contributions and the relative concentration gradient as follows:^1,3^
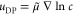
1where *u*_DP_ is the diffusiophoretic velocity, μ̃ is the diffusiophoretic mobility, and *c* is the concentration. The diffusiophoretic mobility depends upon the electrophoretic (μ̃_EP_) and chemiphoretic (μ̃_CP_) contributions.
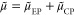
2The electrophoretic contribution originates from the spontaneous electric field, as a result of the diffusion potential gradient that depends upon the ion pair type. This is described by^1,3^
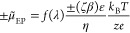
3where *f*(λ) is a size-dependent function, ε is the electrical permittivity, ζ is the surface potential, η is the dynamic viscosity, *k*_B_ is the Boltzmann constant, *T* is the temperature, *z* is the ion valence, *e* is the electron charge, and β is the diffusivity contrast between the anion and cation, defined as
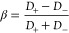
4The chemiphoretic contribution results from the osmotic pressure difference in the Debye layer as a result of the concentration gradient in the bulk^1,3^ and is defined as
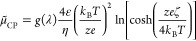
5where *g*(λ) is another size-dependent function. These size-dependent functions [*f*(λ) and *g*(λ)] depend upon λ = (κ*a*)^−1^, which is the ratio of the Debye layer thickness (κ^–1^) and particle radius (*a*). For very thin Debye layers, when the Debye layer is much thinner than the particle radius, so that λ → 0, both *f*(λ) and *g*(λ) approach unity.^1,3^ The equations above are not valid for double layers in the same order of magnitude as the particle radius or much smaller (λ → ∞) because the linearization of the Poisson–Boltzmann equation is not valid under these conditions.^6^

The diffusiophoretic mobility is found by combining eqs 3 and 5 with eq 2, resulting in
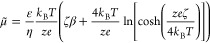
6The diffusiophoretic mobility can be non-dimensionalized by the salt diffusivity; μ = μ̃/*D*_s_, where *D*_s_ is the salt diffusivity defined as follows for monovalent salts:
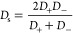
7The magnitude of the diffusiophoresis depends upon the relative concentration gradient (∇ ln *c*), the surface potential (ζ), and diffusivity contrast (β). The magnitude is largest when the electrophoretic and chemiphoretic contributions are in the same direction, where a positive contribution results in movement toward a higher concentration. The chemiphoretic contribution is positive for all ζ potentials, whereas the electrophoretic contribution is positive for a negative ζ potential when *D*_–_ ≫ *D*_+_ or a positive ζ potential when *D*_+_ ≫ *D*_–_. As such, these two contributions can either enhance or reduce the resulting diffusiophoretic motion.

Diffusiophoresis can be measured via particle displacement along a cross-gradient in a co-flow system,^7−9^ via a wall reaction,^10−12^ and via counter-gradients.^13−15^ The focus here is on co-flowing systems in a H-shaped channel. A H-shaped channel is ideal to measure diffusivity of a solute,^16^ and it can also be used to measure diffusiophoresis. The H-shaped channel is a simple microfluidic device in design and operation, such that complex systems can be measured.

Abécassis et al.^7^ studied diffusiophoresis under salt gradients in a Ψ-shaped microchannel, where a colloid solution was fed in the middle channel. They added different salts (NaCl, KCl, and LiCl) either to the colloid solution or outside the colloid solution, causing the colloids to spread or focus. Spreading of the colloids was observed when the salt was added outside the colloid solution, and focusing occurred when the salt was added to the colloid solution. The movement was always directed toward a higher concentration and attributed to diffusiophoresis, which was validated with an analytical and numerical approach. The chemiphoretic contribution was the same for all experiments because the ζ potential was constant and the electrophoretic contribution increased with an increasing diffusivity contrast, such that movement ranked from LiCl (β = −0.33) to NaCl (β = −0.21) to KCl (β = −0.02).

Research toward diffusiophoresis has increased in complexity of the systems over the last decades. For example, Abécassis et al.^7^ studied a basic diffusiophoretic system under salt gradients, and Visan and Lammertink^8^ increased complexity of the system using photocatalytic particles, thus including an *in situ* generated concentration gradient. Other elaborations have been made, for example, on high-salinity gradients,^17,18^ multicomponent systems,^19−21^ and reactive systems (other than Janus particles).^4,22^ However, there are still more simple systems to explore to understand the more complex systems. In particular, electrolytes that possess a strong diffusion contrast or large β values concern class solutes that have been relatively unexplored.

Inorganic salts usually have a diffusivity contrast ranging between −0.4 and 0.4.^11^ Stronger diffusivity contrasts are found for surfactants^23,24^ or strong acids and bases. Strong acids and bases have higher diffusivity contrasts because the diffusivity of a proton and hydroxide are high compared to the other cations and anions.^4,25^ For example, hydrochloric acid (HCl) has a diffusivity contrast of 0.64, and for caustic soda (NaOH), β = −0.60. Carboxylic acids generally have a proton as the cation and a large anion from the carboxylic group, creating even larger diffusivity contrasts.

Here, we investigate diffusiophoretic movement of ordinary polystyrene particles in a H-shaped microchannel under different solute gradients. The systems explored here are first of the well-known salts, lithium chloride and sodium bicarbonate, which are expected to behave according to theory. These salts are used to validate the setup. In the second system, we use the carboxylic acids, formic, acetic, and oxalic acids. Strong acids have been measured before,^18,26^ but these weaker acids offer the opportunity to study larger diffusivity contrasts, because most inorganic salts have −0.4 < β < 0.4.^11^ Lastly, the diffusivity contrast of formic acid is modified by replacing the proton with the anion sodium, potassium, or lithium. These organic formate salts are studied to compare their behavior to the known theory.

## Experimental Section

### Chemicals

The chemicals used during the experiments were lithium chloride (≥99%, Sigma-Aldrich), sodium bicarbonate (99.5–100.5%, Sigma-Aldrich), formic acid (96+%, Alfa Aeser,), acetic acid (99.0–100%, Boom), oxalic acid (98.0%, Sigma-Aldrich), sodium hydroxide (≥98.0%, Sigma-Aldrich), potassium hydroxide (≥85.0%, Sigma-Aldrich), and lithium hydroxide (≥98.0%, Sigma-Aldrich). The compounds are dissolved or diluted with pure water (Milli-Q grade).

### ζ Potential of the Particles

Polystyrene particles functionalized with fluorescence (PS-FluoRed-Fi329) were purchased from Microparticles GmbH. The particles were synthesized by polymerization of polystyrene with potassium persulfate, forming sulfate groups at the surface. Because sulfate is a strong acid (p*K*_a_ ≈ −1.9 of methanosulfonic acid), the particle surface groups were fully dissociated under the experimental conditions. The particles were spherical with a diameter of 1.14 ± 0.03 μm and a density of 1.05 g/cm^3^.

The ζ potential of the particles was measured in the Zetasizer Nano ZS (Malvern Panalytical), which uses laser Doppler velocimetry and phase analysis light scattering to measure the particle electrophoretic mobility. The ζ potential was calculated from the electrophoretic mobility via Henry’s function
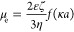
8where μ_e_ is the electrophoretic mobility, ε is the fluid permittivity, η is the dynamic viscosity, and *f*(κ*a*) is Henry’s function, which depends upon the Debye layer (κ^–1^) and particle radius (*a*).^27,28^ This function is valid for weak, constant surface potentials such that the electrostatic potential is described by the linearized Poisson–Boltzmann equation. For our particle diameter of 1.14 ± 0.03 μm, we have a relatively large κ*a* value, suggesting that the surface potential should be below 125 mV.^6^ Henry’s function knows two limits: *f*(κ*a*) → 1 for a thick double layer (κ*a* ≪ 1) and *f*(κ*a*) → ^3^/_2_ for a thin double layer (κ*a* ≫ 1), which is the Smoluchowski mobility. The full expression for *f*(κ*a*) can be found in the study by Swan and Furst.^27^

### Diffusiophoresis Measurements

**Figure 1:**
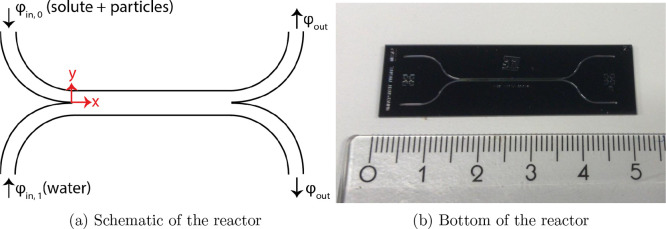
(a) H-shaped reactor shown schematically and (b) actual reactor with a scale in centimeters. The main channel is 600 μm wide, 100 μm high, and 2 cm long.

The experimental measurements were performed in a H-shaped channel, which is schematically shown in Figure 1a, and the actual reactor is shown in Figure 1b. The channel has the dimensions of 2 cm length, 600 μm width, and 100 μm depth, between inlets and outlets.

The microreactor was fabricated in the MESA+ Nanolab Cleanroom from an oxidized p-type silicon wafer and MEMpax wafer. Five reactors were produced from one wafer. The channel was etched in the silicon wafer according to the following steps. First, a positive photoresist (Olin Oir 907-17) was coated on the wafer, excluding the shape of the channel. The channel was etched via deep reactive ion etching (DRIE), where the depth was checked between etching cycles until a depth of 100 μm was achieved. Next, the front side of the wafer was coated with a spray-coating photoresist (AZ4999) and a protection foil (Harke i-HC), so that the main channels were protected. On the backside, a negative photoresist (SU8) was used and the inlets and outlets were prepared using powder blasting. After the powder blasting, all protection layers of photoresist and foil were removed and the silica layer was removed with 1% buffered hydrofluoric acid (BHF). The clean silicon wafer with a channel was bonded to the MEMpax wafer via anodic bonding. Lastly, the wafers were cut into five reactors of 15 × 50 mm.

A reactor was placed in a homemade aluminum reactor holder. The inlets and outlets were connected via polyetheretherketone (PEEK) tubing and connections. A Harvard PHD syringe pump with a push–pull mechanism was used in combination with four glass syringes of 1 or 10 mL (Hamilton T-1000 gas tight syringes with luer lock), which was needed to ensure stable flow without pressure differences over the whole system.

Before the start of each experiment, the reactor was flushed with water (Milli-Q quality) before the solute with particles was introduced into the reactor. The flow rate during flushing was kept at 100 μL/min and reduced to 1 μL/min for the experiment (0.56 mm/s). The solute concentration was 10 mM for each experiment with 0.02 wt % particles, which were sonicated for 5 min prior. The flow was stabilized for 20 min before the microscopy pictures were taken.

The microscope used was a Carl Zeiss Axio Observer Z1 with a LaVision Imager intense camera, in combination with a 5× objective (Epiplan-Neofluar 5×/0.13 HD DIC M27). Images were taken at each location in the channel with a shutter time of 1 ms at 9.84 Hz for 3 s, collecting 30 images. Additionally, a bright-field image was taken to determine the channel locations.

The images collected per locations were first post-processed in ImageJ to increase contrast and then processed in MATLAB to determine the intensity profiles per location. An intensity profile was taken with intervals of 1 mm channel length. The intensity profiles were fitted with a normal cumulative distribution function to determine the particle displacement. A positive displacement (Δ*w* > 0) represents diffusiophoresis toward higher solute concentrations, and a negative displacement (Δ*w* < 0) represents diffusiophoresis toward lower concentrations.

## Numerical Model

The diffusiophoretic movement of particles is studied theoretically in a numerical COMSOL Multiphysics version 5.6 model, with the same channel geometry as shown in Figure 1a. A two-dimensional geometry was used with the same width and length as the experimental channel. The fluid flow through the channel was described by Stokes flow, because the inertial term could be neglected as a result of low Reynolds numbers. The shallow channel approximation was used to account for the channel depth (100 μm). The inlets consisted of fully developed flow with average velocity of 0.56 mm/s, and the outlet boundary condition was at zero static pressure with suppressed backflow.

The species balance was solved with the transport of diluted species module with convection, where the velocity field was coupled to the creeping flow module. The initial concentration in the whole channel was zero, and species were introduced via inlet ϕ_in,0_ (see Figure 1a), with outflows at ϕ_out_.

The particle tracking module was used for particle transport, which were introduced in inlet ϕ_in,0_. It was assumed that the particles were of uniform size, which freeze at the wall, with density of 1050 kg/m^3^ and diameter of 1.14 μm, without a charge. The influence of the charge was included in the diffusiophoretic movement, which was added to the drag force of the particles. The particle velocity (*u*_p_) is the summation of the fluid velocity (*u*_f_) plus the diffusiophoretic velocity (*u*_p_ = *u*_f_ + μ̃∇ ln *c*) in the *x* and *y* directions. For this, the concentration profile from the diluted species calculation was used to define ∇ ln *c* throughout the domain, to define the force on each particle using the constant diffusiophoretic mobility μ̃ from Table 1. The particles were introduced at ϕ_in,0_ from 0.01 to 60 s for every 0.2 s with 50 particles per release and exit at ϕ_out_.

**Table 1:** Particle ζ Potential, Diffusivity Contrast (β), Electrophoretic (μ̃_EP_) and Chemiphoretic (μ̃_CP_) Contribution to the Diffusiophoresis, Diffusiophoretic Mobility Normalized by the Salt Diffusivity (μ), and Direction of the Diffusiophoresis

compound	ζ (mV)	β	μ̃_EP_ (×10^9^, m^2^/s)	μ̃_CP_ (×10^9^, m^2^/s)	μ	direction of diffusiophoresis
acetic acid	–58.5 ± 2.6	0.79	–0.82	0.28	–0.28	lower
formic acid	–59.6 ± 8.4	0.73	–0.77	0.29	–0.19	lower
oxalic acid	–61.2 ± 9.7	0.79	–0.86	0.31	–0.29	lower
lithium formate	–75.5 ± 8.5	–0.68	0.23	0.45	0.56	higher
sodium formate	–72.6 ± 7.8	–0.04	0.06	0.42	0.34	higher
potassium formate	–58.7 ± 4.0	0.15	–0.15	0.28	0.13	higher
lithium chloride	–76.7 ± 8.9	–0.33	0.44	0.47	0.91	higher
sodium bicarbonate	–93.8 ± 8.4	0.06	–0.10	0.67	0.57	higher

The mesh was physics-controlled with a fine element size for all modules, where the mesh size was checked for independency. The velocity and solute concentration profile were first solved in a stationary study, because it reaches a steady-state solution for the specific velocity and diffusion coefficient combination. Second, the particle tracing was solved in a time-dependent study over the range of 0.01–60 s with steps of 0.1 s. The particle positions were further analyzed in MATLAB to determine the maximum particle movement over the length of the channel.

## Results and Discussion

### Particle ζ Potential

The particle ζ potential was measured for all compounds of interest at a concentration of 10 mM. The particle concentration was kept at 0.001 wt % for the salts and 0.02 wt % for the carboxylic acids. The salts were measured with a low particle concentration because that was sufficient to execute the measurement with acceptable measuring accuracy. The particle concentration was higher for the carboxylic acids, because the carboxylic acids form bonds on the particle surface, thus changing the surface charge. Realistic values were found when the particle concentration was matched with the particle concentration in the diffusiophoretic movement measurements. The results are shown in Table 1.

The ζ potential can be divided into roughly three categories depending upon the solution pH: acidic, neutral, and basic. Sodium carbonate solution has a pH of approximately 9, thus slightly basic, and results in the most negative ζ potential for the particle, −93.8 ± 8.4 mV. Sodium formate, lithium formate, and lithium chloride are approximately neutral (pH 6–7), which results in a ζ potential of around −75 mV. Potassium formate was expected to be around neutral pH, but it was measured at pH 4.6, thus slightly acidic, which results in a less negative ζ potential. Lastly, the carboxylic acids result in a ζ potential of around −60 mV, even though the pH ranges from 2.1 (oxalic acid) to 3.4 (acetic acid).

The electrophoretic and chemiphoretic mobilities are calculated from the ζ potential and diffusivity contrast (β) via eqs 3 and 5 and are shown in Table 1. The diffusivity contrast β is based on the cation and anion diffusivities, which are given in the Supporting Information. The data given here are used later on to discuss the experimentally measured diffusiophoresis.

### Lithium Chloride and Sodium Bicarbonate

**Figure 2:**
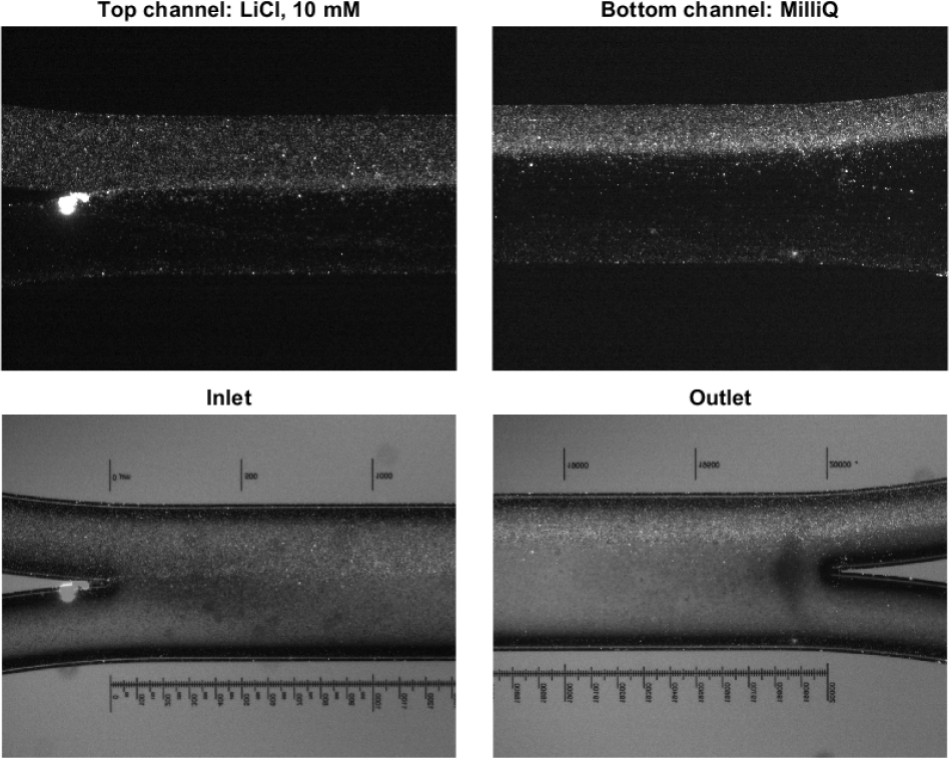
Microscopy images for the experiment with lithium chloride. The top images show the fluorescent particles at the inlet (top left) and outlet (top right). The bottom images are the bright field images overlaid with the fluorescent particles at the inlet (bottom left) and outlet (bottom right).

The diffusiophoretic movement is measured in lithium chloride (LiCl) and sodium bicarbonate (NaHCO_3_) solutions. The microscopy images taken during the experiment are shown in Figure 2. The movement of the particles is clearly visible when the inlet and outlet are compared to each other.

**Figure 3:**
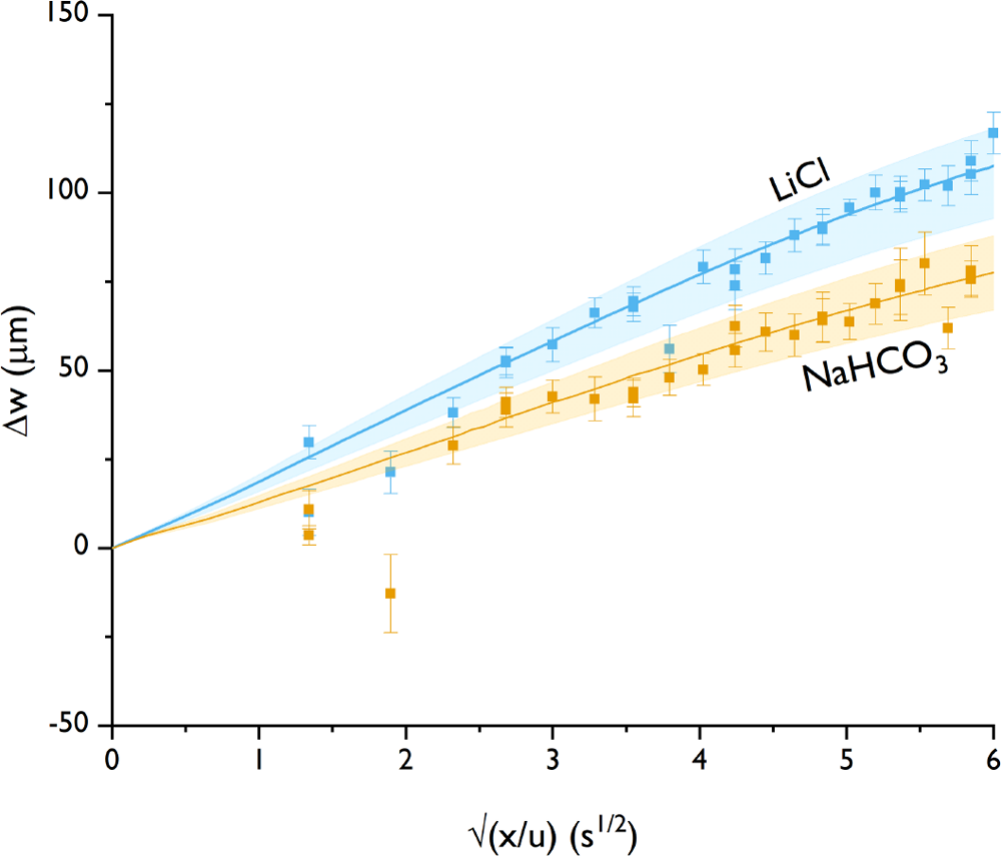
Diffusiophoretic movement of polystyrene particles for 10 mM lithium chloride and 10 mM sodium bicarbonate. The points are the experimental values, and the lines are the numerical model predictions, including error margins.

The diffusiophoretic movement is plotted against , where *u* is the fluid velocity (0.56 mm/s), because then the slope should equal an effective diffusion coefficient.^7,29^Figure 3 shows the diffusiophoretic movement for both the experiments (symbols) as well as the model (lines), where the inlet concentration was 10 mM, with 0.02 wt % polystyrene particles. The input for the model is based on the parameters presented in Table 1 and is thus not fitted to the data.

The diffusiophoretic movement is toward a higher concentration for both LiCl and NaHCO_3_, with good agreement between experimental observations and the model prediction (within the error margins). The out-of-line experimental points are due to some local contaminations within the channel, leading to erroneous position determination.

The diffusiophoretic movement of LiCl is toward higher concentrations because the electrophoretic and chemiphoretic contributions are in the same direction (both are >0). For NaHCO_3_, β is positive because the diffusivity of sodium is higher than bicarbonate (*D*_+_ > *D*_–_). Therefore, the electrophoretic contribution is opposing the chemiphoretic contribution. The movement is still toward higher concentrations because the chemiphoresis dominates the electrophoresis.

From Figure 3, it can be concluded that diffusiophoretic movement of polystyrene particles can be measured under salinity gradients. The experimental results agree well with the numerical model, which is only based on the measured particle ζ potential and diffusivity contrast of the used salt.

### Carboxylic Acids

The diffusiophoretic movement is measured in gradients of formic acid (FA), acetic acid (AA), and oxalic acid (OA). The movement is expected toward lower concentrations, because the proton has a higher diffusivity than the acid, resulting in a dominating electrophoretic contribution toward lower concentrations and an overall negative diffusiophoretic mobility (Table 1).

**Figure 4:**
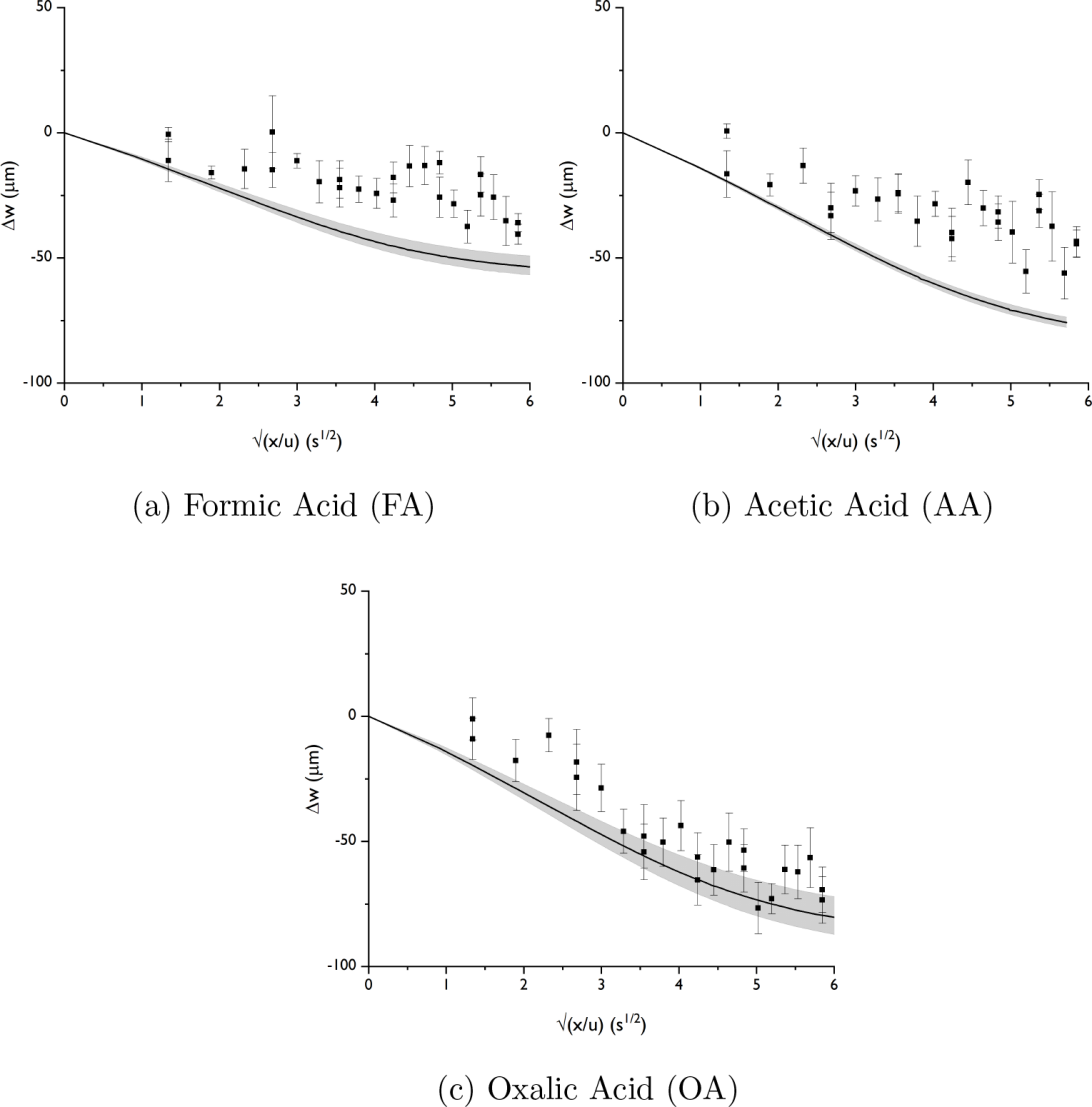
Diffusiophoretic movement of polystyrene particles under formic acid, acetic acid, and oxalic acid gradients.

Figure 4 displays particle displacement toward lower concentrations. The particle velocity increases from FA to AA to OA, as expected from the diffusivity contrast and electrophoretic contribution. However, the displacement measured during the experiments is somewhat less than expected from the numerical model. The results from OA are closest to the numerical model, but for all of them, the experimental displacement is less compared to the model prediction.

The carboxylic acids are all weak acids, meaning that only a portion of them is dissociated, as seen in Table 2. Only 1.2 mM of 10 mM is dissociated of FA. Although this does not influence the relative gradient, the diffusiophoretic mobility (μ̃) can decrease with the salt concentration,^19^ because it depends upon the Debye layer thickness and ζ potential, which both depend upon the solute concentration. The Debye layer thickness is 9 nm at 1.2 mM salt concentration; therefore, the derivation of the diffusiophoretic velocity is still valid for the experimental conditions.

**Table 2:** Amount of Dissociated Carboxylic Acids Based on the Inlet Concentration and pH^a^

acid	p*K*_a_	pH_inlet_	*c*_dissociated_ (mM)
formic acid	3.75	3	1.2
acetic acid	4.76	3.4	0.4
oxalic acid	1.27	2.1	8.6

a The calculations are shown in the Supporting Information.

**Figure 5:**
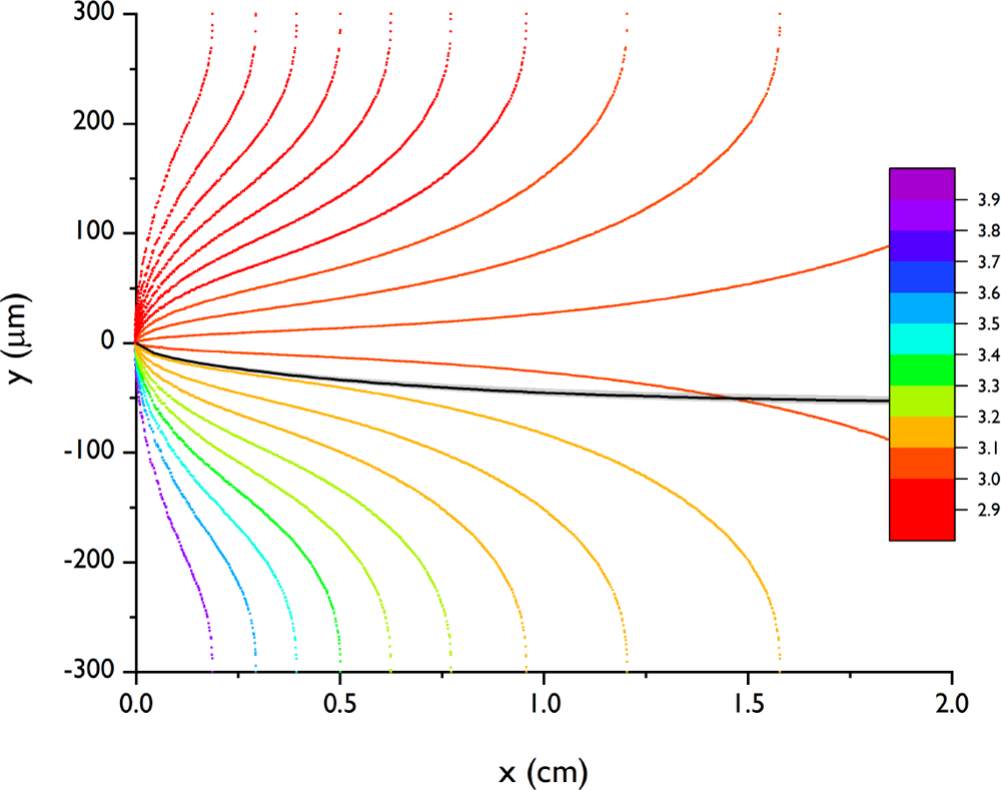
Influence of pH over the length and width of the channel for formic acid. The black line represents the maximum particle movement according to the numerical model. The particles are introduced at the top channel (0 < *y* < 300 μm).

The pH does not change significantly over the range of 5–10 mM for all carboxylic acids, resulting in a relatively stable particle ζ potential (see the Supporting Information). However, the pH gradient is very significant at the inlet, where carboxylic acid and water streams meet. The diffusiophoretic movement depends upon the local pH via the particle ζ potential. The pH distribution of FA in the channel is determined via the concentration of FA and calculated via the equations given in the Supporting Information and shown in Figure 5.

The pH changes the most in the first 5 mm of the channel, where the pH quickly drops from neutral to approximately 4 on the water side (between *y* = −300 and 0 μm) and then decreases gradually further to approximately 3. The particles are located in the acid solution; therefore, the pH around the particle front changes between 2.9 (inlet) and 3.2 over the whole length of the channel. Even though there is a large pH gradient at the inlet of the channel, the particle ζ potential is not significantly influenced by these local gradients.

From the Debye layer thickness and relatively stable ζ potential, it can be concluded that the diffusiophoretic mobility does not change significantly during the experiment using FA. OA is a stronger acid compared to FA; therefore, 8.6 mM dissociated at pH 2.1 (see Table 2). AA is the weakest acid, where a pH of 3.4 corresponds to 0.4 mM dissociated acid and a Debye layer thickness of 21 nm. Even though the carboxylic acids are weak acids, which are partly dissociated, this does not significantly change the Debye thickness or particle ζ potential at the used concentrations.

Abécassis et al.^7^ showed that salt concentrations of 10 mM and larger are needed to obtain the maximum diffusiophoresis. At lower concentrations, the effect of the buffer becomes more relevant and lowers the effective diffusiophoresis. Nery-Azevedo et al.^23^ encountered a similar issue when measuring the diffusiophoretic movement of latex colloids under surfactant gradients. The theory overpredicted the diffusiophoretic movement 2–3-fold for weak applied gradients (Δ*c* = 2 mM), whereas larger gradients (Δ*c* = 4–6 mM) were closer to the theory. Gupta et al.^18^ studied the influence of the concentration on the diffusiophoresis in a dead-end pore by changing the boundary conditions in a numerical study (constant current versus constant potential) and comparing it to experimental results. For NaCl, their movement was approximately described by the constant current boundary condition for 1–10 mM, but for 0.1 mM, the movement cannot be described by the constant current or potential boundary conditions and the charge regulation boundary condition would be most suitable.

Here, the movement is closest to the theory when most acid is dissociated (oxalic acid), with less movement when less acid is dissociated. The magnitude of diffusiophoresis depends upon the relative concentration gradient; however, a limitation of the absolute gradient is observed here. This limitation is not predicted by the theory within the ranges researched here, because the mobility is concentration-dependent but is considered stable over the range.

### Formate Salts

**Figure 6:**
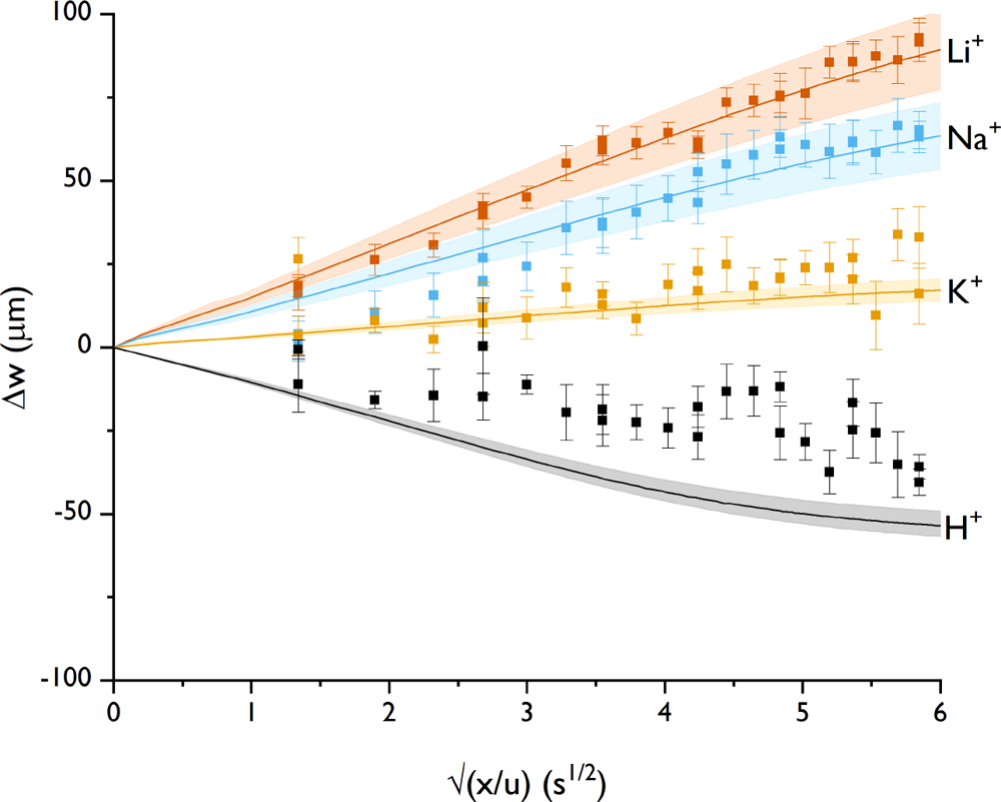
Influence of the cation of formate on the diffusiophoretic movement, where Li^+^ represents lithium formate, Na^+^ represents sodium formate, K^+^ represents potassium formate, and H^+^ represents formic acid (same as Figure 4).

Formic acid was neutralized with either lithium hydroxide, sodium hydroxide, or potassium hydroxide to obtain the organic salts lithium formate (LF), sodium formate (SF), or potassium formate (PF). The pH was around neutral for all of these solutions. The results are compared to FA and shown in Figure 6.

The movement of all formate salts is toward higher concentrations, as expected from Table 1, with FA toward lower concentrations. The differences were discussed in the previous section between the experimental and theoretic movements of FA. The chemiphoretic and electrophoretic contributions are in the same direction for LF and SF, although, for PF, the electrophoretic contribution is opposing the chemiphoretic contribution. The trend Li^+^ > Na^+^ > K^+^ is observed for diffusiophoretic movement. This trend is explained by the diffusivity contrast, which is most negative for LF, slightly negative for SF, positive for PF, and even more positive for FA. The same trend was observed by Abécassis et al.,^7^ who measured diffusiophoresis under chloride salt gradients. The experiments here show that organic salts behave similar to inorganic salts.

## Conclusion

Diffusiophoretic movement of ordinary polystyrene particles can be measured in H-shaped microchannels under different solute gradients. It was shown that the theory and experiments are in accordance with each other for ordinary inorganic salts and organic formate salts. Carboxylic acids did not cause the diffusiophoretic movement, which was expected from the theory, because they are partially dissociated and the ionic strength is less compared to the inorganic salts. The relative gradient and mobility remained unaffected by this decrease in ionic strength.

The H-shaped microreactor has shown to be a simple platform to measure diffusiophoretic movement under different solute gradients. This platform could be used in future endeavors for more complex systems, like the influence of mixed ion gradients, minimum absolute gradient, or reactive systems. The combination with the numerical model ensures deeper insight into the system studied, like the true concentration and pH gradients. Additionally, the numerical model could be used to fit the experimental results to extract an effective diffusion coefficient.

## Supporting Information Available

Diffusion coefficients of the different ionic species, with their ambipolar diffusivity and diffusivity contrast, surface ζ potential measured for concentrations ranging from 5 to 10 mM of formic, acetic, and oxalic acids, and equations given to calculate the pH and amount of dissociated acid based on the dissociation constant and inlet concentration (PDF)
