# Supplementary material for: Diffusiophoretic Movements of Polystyrene Particles in a H-Shaped Channel for Inorganic Salts, Carboxylic Acids, and Organic Salts
Source: Langmuir. 2022 Sep 28;38(40):12140–7. doi: 10.1021/acs.langmuir.2c01577 (PMC9558484; doi:10.1021/acs.langmuir.2c01577)
Supplement: Supplementary file 1 — la2c01577_si_001.pdf [file la2c01577_si_001.pdf]

Diffusiophoretic Movements of Polystyrene Particles in an H-Shaped Channel for Inorganic Salts, Carboxylic Acids and Organic Salts

Nicole A.B. Timmerhuis and Rob G.H. Lammertink*

Soft Matter, Fluidics and Interfaces, University of Twente, MESA+ Institute for Nanotechnology, P.O. Box 217, 7522 NB, Enschede, the Netherlands

E-mail: r.g.h.lammertink@utwente.nl

Supporting Information Available

Diffusivity constants

The diffusivities of all measured compounds are given in Table S1.

Table S1: Diffusivities of the compounds[1] with the respective ambipolar solute diffusivity D_s and the diffusivity contrast β

Compound	D_+ 10^{-9} [m^2/s]	D_- 10^{-9} [m^2/s]	D_s 10^{-9} [m^2/s]	β
Acetic acid	9.31	1.09	1.95	0.79
Formic acid	9.31	1.45	2.52	0.73
Oxalic acid	9.31	1.07	1.92	0.79
Sodium formate	1.33	1.45	1.39	-0.04
Lithium formate	1.03	1.45	1.20	-0.17
Potassium formate	1.96	1.45	1.67	0.15
Lithium chloride	1.03	2.03	1.37	-0.33
Sodium bicarbonate	1.33	1.18	1.25	0.06

Particle ζ-potential for the carboxylic acids

The ζ-potential is measured over a range of concentrations for the carboxylic acids. The results are shown in Figure S1.

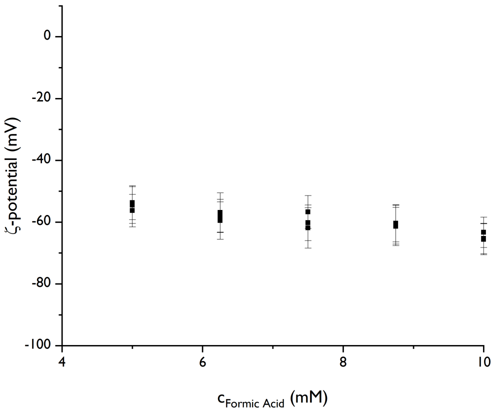

(a) Formic Acid (FA)

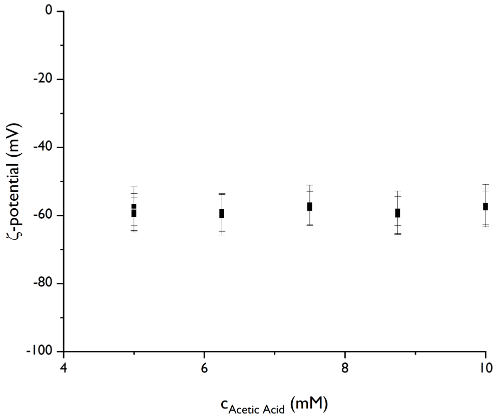

(b) Acetic Acid (AA)

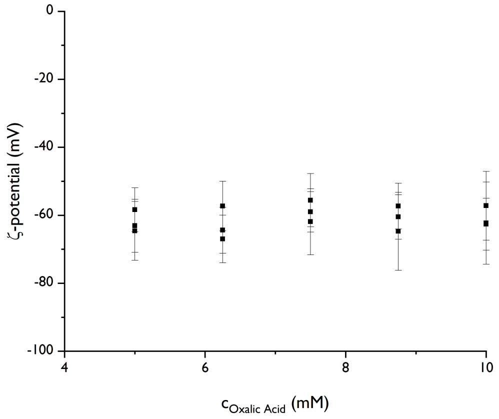

(c) Oxalic Acid (OA)

Figure S1: The particle ζ-potential as a function of the acid concentration between 5 and 10 mM.

The particle ζ-potential is constant for all measured concentrations of carboxylic acids. Over a range of 5 till 10 mM, the pH changes for formic acid from 3.1 to 3, for acetic acid from 3.6 to 3.4, and for oxalic acid from 2.4 to 2.2. It is expected that the ζ-potential mostly depends on the solution pH. The pH does not change in the range of 5 till 10 mM for all carboxylic acids, and there is also no significant change in ζ-potential.

Calculate pH of the acids

The amount of dissociated acid is determined by calculating the pH of a weak acid solution based on the inlet concentration and pK_a-value. We consider a weak acid with concentration [HA] in mole/L, which dissociates in water.

$$[\mathrm{HA}] + [\mathrm{H_2O}] \rightleftharpoons [\mathrm{A^-}] + [\mathrm{H_3O^+}] \tag{S1}$$

The equilibrium is determined by the acid dissociation constant

$$K_\mathrm{A} = \frac{[\mathrm{A^-}][\mathrm{H_3O^+}]}{[\mathrm{HA}]} \tag{S2}$$

where the acid dissociation constant is related to pK_a according to $pK_a = -\log_{10}(K_\mathrm{A})$.

The total concentration acid (c_{HA}) is the amount of dissociated and neutral compound together

$$c_{HA} = [\mathrm{HA}] + [\mathrm{A^-}] \tag{S3}$$

Lastly, the total charges in the system should be equal to zero in order to maintain electroneutrality

$$[\mathrm{H_3O^+}] - [\mathrm{A^-}] = 0 \tag{S4}$$

Equations (S2) and (S3) are combined to describe $[\mathrm{A^-}]$ as a function of K_A, c_{HA}, and $[\mathrm{H_3O^+}]$

$$[\mathrm{A^-}] = \frac{K_\mathrm{A} c_{HA}}{[\mathrm{H_3O^+}] + K_\mathrm{A}} \tag{S5}$$

According to Eq. S4, $[\mathrm{H_3O^+}] = [\mathrm{A^-}]$, resulting in

$$[\mathrm{H_3O^+}]^2 + K_\mathrm{A}[\mathrm{H_3O^+}] - K_\mathrm{A} c_{HA} = 0 \tag{S6}$$

which can be solved according to the ABC-formula to obtain the concentration of $[H_3O^+]$

$$[H_3O^+] = [A^-] = -\frac{K_A}{2} + \frac{1}{2}\sqrt{K_A^2 + 4K_A c_{HA}} \tag{S7}$$

Lastly, the pH is calculated as following pH$= -\log_{10}([H_3O^+])$. These equations are applicable to all the carboxylic acids discussed.

References

(1) aqion, Table of Diffusion Coefficients. 2020; https://www.aqion.de/site/194.
